# A special issue on DNA damage response and genome stability

**DOI:** 10.1186/2045-3701-2-4

**Published:** 2012-02-27

**Authors:** Guo-Min Li

**Affiliations:** 1 Markey Cancer Center, Graduate Center for Toxicology, University of Kentucky College of Medicine, Lexington, KY

## 

The fidelity of the genome is under constant threat by exogenous and endogenous reactive species, including toxic chemicals, ionizing radiation and byproducts of normal cellular metabolism. These species cause damage to DNA by modifying DNA bases, breaking DNA strands and/or altering DNA structures. When this happens, the consequences to the cell can be disastrous, ranging from single gene mutations to massive chromosomal breakdown and rearrangements. These instabilities lead to severe human diseases including cancer.

Fortunately, humans and other eukaryotes have evolved multiple mechanisms to ensure genome stability. These mechanisms can be classified into two major systems, one that deals with replication fidelity and the other that takes care of various DNA lesions. The replication fidelity maintenance system involves DNA polymerases and the DNA mismatch repair pathway. First, the 3'-5' proofreading nuclease activity of replicative DNA polymerases is responsible for removing the majority of mis-incorporated nucleotides during DNA replication [1]. However, there are always cases where certain mis-incorporations escape from the proofreading activity. In the latter situation, the mismatch repair machinery [2] is recruited to the site to correct the mismatch. If a DNA template contains a bulky DNA adduct that blocks the polymerization activity of replicative polymerases, a specialized DNA polymerase, also called translesion synthesis polymerase, is employed to bypass the bulky DNA lesion, followed by a switch back to the replicative polymerase to continue normal replication [3].

The DNA damage processing system can be divided into two steps: DNA damage response and DNA repair. Extensive studies in the past 20 years have elucidated the mechanisms of several major DNA repair pathways that remove almost all kinds of DNA lesions. These pathways include base excision repair [4], nucleotide excision repair [5,6], mismatch repair [2] and double strand break repair [7,8].

DNA damage response is a complex signal transduction process that has the ability to sense DNA damage and transduce the information to the cell to direct cellular responses to the damage. An arsenal of protein activities has been identified, which function to be damage sensors, mediators, transducers and effectors during DNA damage response [9]. Unlike the DNA repair pathways, however, the mechanism of DNA damage response is much less known.

In this issue, *Cell & Bioscience *presents a series of reviews attempting to define the most challenging questions in DNA damage response and repair, and to provide an overview of the latest breakthroughs and developments in the field. The article by Nan Wu and Hongtao Yu [10] explores the mechanism by which the structural maintenance of chromosomes (Smc) proteins and non-Smc proteins, which are required for chromatid cohesion, chromosomal segregation and condensation, regulate DNA damage response and repair. A second article by Bin Wang [11] describes how BRCA1, an important tumor suppressor and a critical DNA damage response mediator, interacts with its partners to regulate activities of multiple repair and checkpoint pathways for genome maintenance. Maintaining the stability of repetitive DNA sequences, particularly the (CAG)_n _and (CTG)_n _trinucleotide repeats, whose expansions cause numerous human disorders, is extremely challenging, as is the understanding of the mechanisms that regulate the stability/instability of the repetitive sequences. In their article, Guoqi Liu and Michael Leffak [12] debate how collaborative efforts by the replication machinery and multiple DNA repair pathways stabilize (CAG)_n _and (CTG)_n _trinucleotide repeats.

It is our sincere hope that this special issue brings our readers enlightenment and offers sufficient introductory information to help them appreciate new breakthroughs in the field.
